# Molecular and Neurobiological Imbalance from the Use of Technological Devices During Early Child Development Stages

**DOI:** 10.3390/children12070909

**Published:** 2025-07-10

**Authors:** Roberta Rizzo, Gaia Fusto, Serena Marino, Iside Castagnola, Claudia Parano, Xena Giada Pappalardo, Enrico Parano

**Affiliations:** 1 Department of Biomedical and Biotechnological Sciences (BIOMETEC), University of Catania, 95124 Catania, Italy; rizzo.roberta@studium.unict.it (R.R.); gaia.fusto@studium.unict.it (G.F.); xena.pappalardo@irib.cnr.it (X.G.P.); 2 Department of Medical, Surgical Sciences and Advanced Technologies “GF Ingrassia” (DGFI), University of Catania, 95124 Catania, Italy; serena.marino7@studium.unict.it; 3 Co.Re.Com, Regional Committee for Communications, 00193 Rome, Italy; icastagnola-cons@regione.lazio.it; 4 Department of General Surgery and Medical-Surgical Specialties, University of Catania, 95124 Catania, Italy; claudiaparano@pec.ordinemedct.it; 5 Institute for Biomedical Research and Innovation (IRIB), Italian National Research Council (CNR), 95123 Catania, Italy

**Keywords:** epigenetics, childhood, digital device, neurodevelopment, screen use, techno-stress

## Abstract

**Background/Objectives:** Digital technologies have become increasingly integrated into the daily lives of children and adolescents, largely because their interactive and visually engaging design is particularly suited to the younger users. The COVID-19 pandemic further accelerated this trend, significantly lowering the average age of access to the digital devices. However, scientific consensus remains divided regarding the developmental impact of digital media use—particularly its cognitive, motor, and emotional consequences—depending on whether the use is passive or active. This review aims to explore these effects across developmental stages, focusing on both behavioral and neurobiological dimensions, and to identify emerging risks and protective factors associated with digital engagement. **Methods**: A PRISMA review was conducted on the impact of digital media use among pre-school children and adolescents. Behavioral, psychosocial, and neurobiological aspects were examined, with specific attention to epigenetic changes, techno-stress, digital overstimulation, and immersive technologies (e.g., virtual and augmented reality). **Results**: The findings suggest that passive digital consumption is more often associated with negative outcomes, such as impaired attention and emotional regulation, especially in younger children. Active and guided use may offer cognitive benefits. Neurobiological research indicates that chronic exposure to digital stimuli may affect stress regulation and neural development, possibly via epigenetic mechanisms. Effects vary across developmental stages and individual vulnerabilities. **Conclusions**: A nuanced understanding of digital engagement is essential. While certain technologies can support development, excessive or unguided use may pose risks. This review provides age-specific recommendations to foster balanced and healthy technology use in children and adolescents.

## 1. Introduction

In the rapidly evolving digital era, children are being introduced to screen-based technologies at increasingly younger ages, raising critical questions about their impact on neurodevelopmental trajectories. According to a study conducted by the Pew Research Center in 2020 [1], 98% of children aged 0–6 years have access to electronic devices, with an average exposure of about 1–2 h per day. Research has demonstrated both potential benefits and risks associated with digital device use during key developmental stages [2]. On one hand, interactive and educational apps have been linked to improvements in fine motor coordination, visual-spatial attention, and even emergent literacy when used with parental guidance [3]. On the other hand, growing concerns have emerged around excessive and unsupervised screen exposure, particularly in early childhood, due to its associations with delays in language acquisition, impaired motor control, disrupted sleep, and behavioral dysregulation [4]. These divergent findings highlight the need to investigate not only the quantity but also the quality and context of screen media use. This rise is largely attributed to the COVID-19 pandemic and its social consequences, which have not only increased the overall use of digital devices but also significantly lowered the average age of first access [5,6]. Over 62% of pre-adolescents aged between 11 and 13 already possess one or more social network accounts, contrary to prevailing legal restrictions on their access to digital devices. This early and extensive familiarity with digital technologies is one of the factors promoting the development of the so-called “hyperconnected childhood” where digital interaction starts to influence learning, feelings, and socialization from an early stage. Whereas digital devices may provide educational and social opportunities, unmoderated or excessive use, particularly at key windows of brain development, raises increasing concern. Various global bodies, including the World Health Organization (WHO) and the American Academy of Pediatrics (AAP), advise no screen use at all in infants less than 1 year and no more than an hour of screen time per day at the ages of 2 to 4 [7,8]. Although these standards are not followed well, the long-term impact of early and extended use of digital devices is yet to be discovered.

Despite the mounting evidence, critical gaps persist. Most studies focus on early childhood (ages 0–5), often neglecting middle childhood (6–11 years), which is a period marked by significant neuroplastic changes and socio-cognitive consolidation. Moreover, adolescence, although heavily engaged with digital media, is often treated as a homogeneous category, overlooking distinctions between early, mid, and late stages. This review addresses these gaps by offering an age-stratified synthesis of how digital media affects neurodevelopmental domains, specifically motor skills, cognition, language, emotional regulation, and sleep, while mapping their underlying neurological and epigenetic mechanisms [9,10].

The originality of this review lies in its developmentally anchored framework, which systematically examines how screen media use interacts with neurobiological maturation across distinct age groups. Furthermore, it investigates the epigenetic underpinnings of digital media-induced alterations, including the role of oxidative stress, neuroinflammation, and endocrine disruption—mechanisms increasingly recognized as critical in early-life programming of cognitive and behavioral outcomes [11]. Foundational theories by Lenneberg (1967) [12] on the critical periods of brain maturation and Krashen (1985) [13] on comprehensible input and affective filters provide a theoretical scaffold for interpreting the impact of digital input on language acquisition and emotional development in screen-dominant contexts.

This review also draws on key milestones in developmental neuroscience to contextualize its findings: epigenetic plasticity in early life [14], motor learning and brain connectivity in early childhood [15], neuroendocrine modulation and stress sensitivity [16], the role of BDNF in cognitive flexibility [17], and the interaction between early experience and brain architecture [18].

By synthesizing behavioral outcomes with neuroimaging and epigenetic markers, our aim is to clarify the developmental windows of vulnerability and opportunity, guiding age-specific recommendations for digital media use. In doing so, this work contributes a distinct, interdisciplinary perspective to a field often fragmented by siloed approaches, and provides practical insights for clinicians, educators, and policymakers seeking to safeguard child and adolescent development in the digital age.

**Figure 1:**
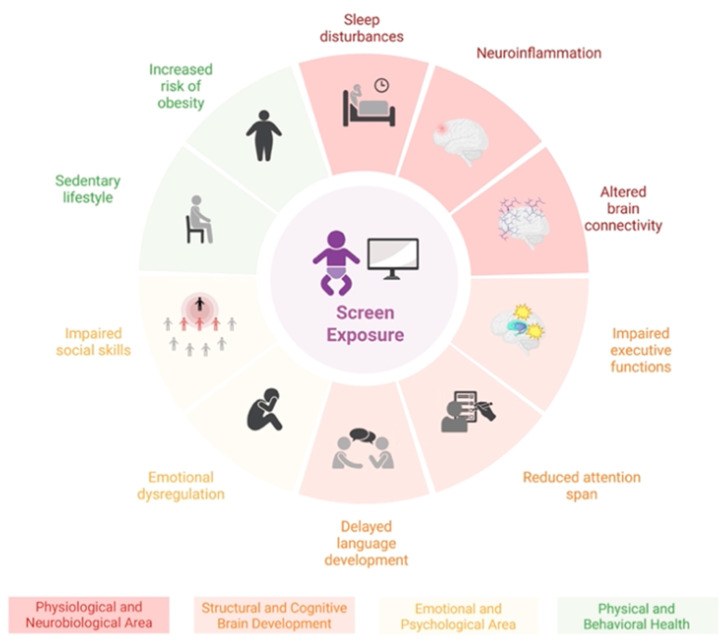
The main consequences of excessive screen time are visually represented and categorized into four thematic areas, each identified by a different color. The layout shows a progression from sleep and brain changes to emotional issues and social or lifestyle impacts in children and adolescents.

This review offers an updated summary of the neurobiological, psychosocial, and behavioral effects of digital device exposure during early childhood (Figure 1).

This is based on the use of new data from epidemiology and advances from the science of neuroscience to examine how screen exposure affects the development of the body and brain, learning, language ability, sleep, emotional function, and mental well-being at all stages of childhood and adolescence.

## 2. Material and Methods

This systematic review was conducted in accordance with the PRISMA 2020 (Preferred Reporting Items for Systematic Reviews and Meta-Analyses) guidelines (https://www.prisma-statement.org/, accessed on 8 January 2025). A comprehensive literature search was performed across PubMed, Scopus, and Web of Science databases to identify peer-reviewed studies published up to May 2025.

The search strategy included combinations of keywords such as “screen time”, “technology use”, “early childhood development”, “neurobiology”, “molecular mechanisms”, and “digital devices”. Moreover, studies were categorized based on clearly defined age groups to ensure consistency in data synthesis and interpretation. The following developmental stages were adopted: infancy and toddlerhood (0–2 years), preschool age (3–5 years), middle childhood (6–11 years), and adolescence (12–18 years). This classification allows for a focused analysis of the impact of electronic device exposure on distinct specific cognitive and motor skills developed during the different neurocognitive and motor developmental phases as established by [12,13]. Both experimental and observational studies, including cross-sectional, cohort, and neuroimaging-based research, were considered. Exclusion criteria included non-English publications, editorials, and studies without primary data, as well as articles with insufficient outcomes of clinical impact, unspecified age of sample size, children with neurodevelopmental disorders or with special needs, a lack of detailed methodological investigation into Information and Communication Technology (ICT) exposure.

**Figure 2:**
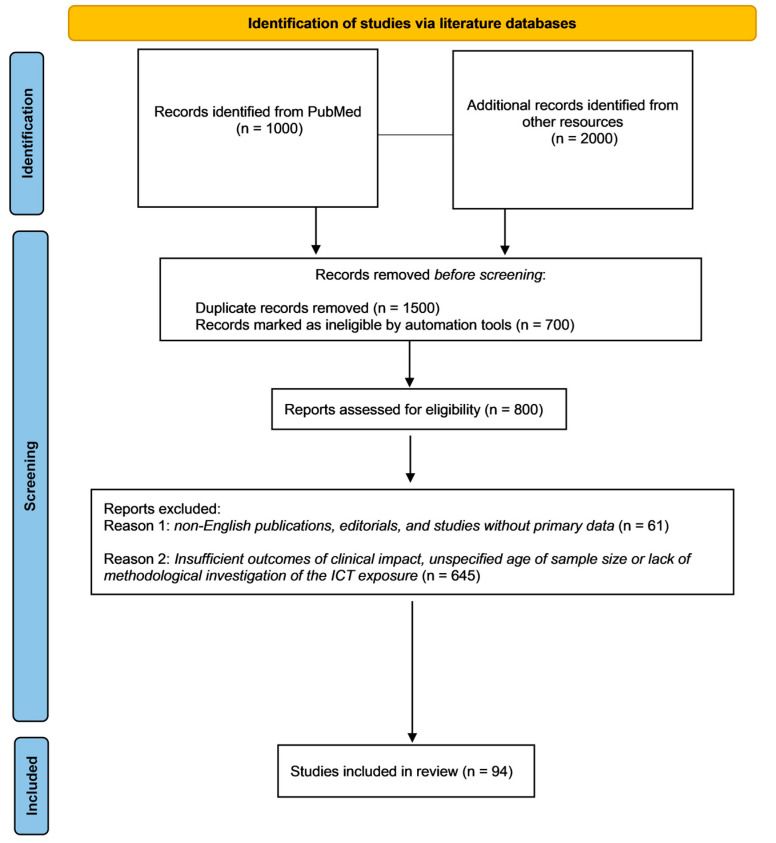
Flowchart of search strategy and selection of reports.

The selection process involved in dependent screening by two reviewers of titles, abstracts, and full texts, with discrepancies resolved by consensus. Data extraction focused on study design, sample characteristics, type and duration of exposure, outcomes measured, and molecular or neurobiological findings (Figure 2).

## 3. Motor Ability

Technology use can have both positive and negative effects on motor skill development. One conducted by Swider-Cios et al. [3] showed a positive association between moderate use of educational applications on tablets and an improvement in motor skills from early childhood (0–5 years). According to other evidence, targeted use of children with interactive content, supervised by parents, appears to contribute positively to motor coordination, navigation and visuospatial skills in 2–3 years toddlers playing with the shape matching app and a storybook apps [19]. This result was also supported by a 12-week teacher-implemented program in 233 preschoolers aged 5–6 years, half working exclusively with paper and pencil, and half partially undertaking their handwriting training with the digital notebook [20]. In this case, the benefits of training with app were most evident among pupils who had the lowest initial scores. These students showed significant improvements in writing performance, as measured by the transfer task [20]. These findings represent qualitative analyses on the modality of digital device use characterized by “active/educational” and possible parent/caregiver involvement during the use. In fact, the excessive use of an electronic device may impair the acquisition of fine motor skills in the under-age-3 child [21,22,23]. A study involving 185 children aged 3 to 6 revealed that unsupervised or non-educational use of digital tools was associated with poorer fine motor performance, particularly in grapho-motor skills and manual coordination, among those who began using digital devices at an earlier age [5]. Similarly, Lin et al. [24] established that excessive use of an interactive touch screen tablet may impair the pre-school child’s fine motor ability. The interactive touch screen, when used intensively, may lack the same motor stimulation as an interactive game which may impair the acquisition of motor skills. The same concept was advanced by Mohamed et al. [25], in which they noted that the motor skills of preschoolers (3–5 years) who do not use touch screens are better than those of children who use a touch screen. Again, according to Arabiat et al. [26], there is a negative correlation between the intensive use of electronic devices by children under the age of 6 and the delay in the development of basic motor skills. Excessive exposure to screen device appears to contribute to a decrease in physical and motor activities, impairing children’s ability to acquire essential motor skills. More, specifically, in preschool children (3–5 yrs), exceeding one hour of daily screen time, especially during evening hours, has been linked to poorer performance in gross and fine motor tasks, as well as postural control [27]. Notably, increased screen exposure in this age group has been associated with a higher risk of suspected developmental coordination disorder [27].

Most research tends to concentrate on children up to age 6, often grouped with preschoolers, while information on children aged 7 to 11 is frequently overlooked or generalized from studies involving mixed-age or mixed-grade populations, often contrasted with adolescents. This gap may stem from the common assumption that the most sensitive periods for neural plasticity and developmental vulnerability occur during early childhood and adolescence [28]. For middle childhood, however, evidence on the relationship between digital tool use and motor development is mixed. On the positive side, interactive digital devices, such as tablets and video games, can enhance fine motor skills and hand-eye coordination [26]. Tasks that require precise finger movements or navigating interfaces can improve dexterity and strengthen the connection between visual input and physical action. Additionally, some technology-based activities stimulate cognitive functions like problem-solving and decision-making, which can indirectly support motor development [26]. On the negative side, in the school-age group (6–10 years), higher exposure to smartphones and tablets correlates with decreased physical activity and poorer sleep quality, factors known to interfere with motor coordination and cognitive recovery processes [2]. It also can contribute to a sedentary lifestyle (see more details in Section 10) that negatively impacts gross motor skills, balance, and coordination. Moreover, overreliance on screens may impair fine motor development, particularly in tasks involving manual dexterity, such as writing or manipulating small objects. It may also disrupt natural hand-eye coordination by shifting focus away from physical interactions with the environment [26].

Instead, studies regarding the adolescent populations do not distinguish effects among early, mid and late adolescence. However, one scoping review found positive evidence in tweens (8- to 12-year-olds) and teens (13- to 18-year-olds) between digital engagements and visual-spatial attention/visual-motor integration, but also in cognitive development, social-emotional development, and mental health and well-being [29] On the contrary, although there are no studies directly focused on motor development skills, most available data express a negative view of excessive technology use among adolescents, highlighting its adverse effects on physical and psychological well-being [4,6,9,10]

## 4. Cognitive and Behavioral Ability

Emerging evidence suggests a potential association between excessive screen exposure and neuroinflammatory responses in children and adolescents that affect the cognitive and behaviour development [9,10]. Notably, current studies have not reported any positive or neutral effects in this context. The observed inflammatory processes may involve the activation of immune cells such as microglia and astrocytes, which are known to play roles in neuroinflammation [4]. Although the long-term implications are not yet fully understood, such processes have been hypothesized to contribute to mood disturbances, cognitive-behavioural difficulties, and possibly increased vulnerability to neurodegenerative conditions [30]. Indeed, based on documented effects of excessive screen time among individuals born after 1980, namely Millennials and Generation Z, it has been estimated that the rates of Alzheimer’s disease (AD) and related dementias could increase by a factor of four to six after the year 2060 [30].

Negative effects on cognitive functions such as attention, memory, learning, and comprehension may have consequences for academic performance. These aspects will be further explored in Section 5.

It has been widely documented that the radiofrequency electromagnetic radiation (RF-EMR) emitted by mobile phones and other wireless devices triggers oxidative stress in various tissues, but also that it causes significant changes in levels of blood antioxidant markers [31]. Possible concerns underlying neural and molecular mechanisms for the behavioral effects in early developmental stage have been proposed in the light of available evidences from the literature in humans and other mammals (prevalently rodents) as reviewed in [32] and reported in research paper [33].

Sage and Burgio [11] were the first to suggest that RF-EMR exposure may affect neurodevelopmental trajectories of the developing brain through epigenetic mechanisms, including DNA methylation, histone modification, and microRNA (miRNA) regulation. During early childhood, heightened brain plasticity (i.e., significant changes in gray and white matter) combined with the structural vulnerability of the developing nervous system (i.e., immature cortical networks, thinner skulls) may prompt adaptive responses aimed at counteracting the potential effects of RF-EMR exposure. This prolonged and harmful early life stress (ELS), also called ‘techno-stress’, has been associated with increased oxidative response, impaired synaptic plasticity, and disruptions in neurotransmission and neuroendocrine system, underlying the development of cognitive and behavioral anomalies [11]. By giving that ELS, particularly maternal stress during the prenatal and postnatal periods, can have lasting impacts on physiological and psychological health of the offspring through epigenetic mechanisms [34,35] one of the largest population-based studies to examine the associations of prenatal-only, postnatal-only, and combined prenatal and postnatal cellphone exposure with emotional and behavioral difficulties is the Danish National Birth Cohort study (2016) [36]. According to these findings, that both prenatal and postnatal exposures may be associated with increased risks of emotional and behavioural difficulties assessed in children (*n* = 51,190) at age 7 years and then at age 11 years [36].

Another proposed pathway involves disruption of circadian rhythms, potentially triggered by prolonged exposure to blue light emitted by screens, which may indirectly influence neurophysiological health [37]. It disrupts the secretion of melatonin, decreases the quality of sleep, and initiates oxidative stress. These interruptions weaken protective antioxidant systems, such as the pathway of the protein known as NRF2 (Nuclear Factor Erythroid 2-related factor 2) and its related targets [38] which play a key role in the cellular response to redox imbalance. Additionally, the blue light emission can trigger neuroimmune responses that may lead to structural changes in the brain, including reduced connectivity between the amygdala and the prefrontal cortex [4,6]. In fact, according to a preprint study [39], adolescent mice (post-natal day 30) exposed to evening blue light displayed increased avoidance behaviors compared to those exposed to darkness or reduced blue light conditions. These findings identify that evening blue light exposure during puberty as a potential risk factor for affective disorders [39].

Apart from this study, neurobiological effects on adolescent population have not been investigated although electronic devices including smartphones, tablets, and laptops have become ubiquitous in adolescent and young adult life. The majority of findings addressed the neurodevelopmental susceptibility most pronounced in childhood even because the first access to technology starts always sooner as preschool-aged children’s. A Functional Near-Infrared Spectroscopy (fNIRS) study [40] indicated that children aged as young as 3 showing high exposure to screens have diminished inhibitory control and under activation of the prefrontal cortex. Other research correlates high levels of screen use with increased levels of cortisol, poorer academic performance, and lower language skills in children in contexts of social vulnerability [13]. In preschool-aged children (3–5 yrs), the screen use beyond 1 h/day, especially in the evening, is significantly linked to modification of neurobiological markers such as reduced levels of BDNF (Brain-Derived Neurotrophic Factor), relevant to support growth, survival, and maintenance, as well as altered white matter integrity [27]. These effects appear to be particularly pronounced in children (*n* = 1026) with limited social interaction (e.g., without siblings), highlighting the vulnerability of this age group (from 1 to 4 years) to the sensory [41]. Parent–child reading time was assessed at age 3, magnetic resonance imaging (MRI) scans were conducted at age 6, and socio-emotional competence was evaluated at age 7 [41].

## 5. Learning and Memory Ability

Despite extensive evidence for children’s education through apps and devices, it still remains to be established to what extent they really learn to apply it in real life. Young children, as well as children younger than 3 years old, are known not to learn as much as from real life interaction and tend not to apply what they have learned through videos to real life situations, a fact which has also been referred to as the ‘video deficit’ [42]. The ‘video deficit’ is a well-documented phenomenon observed between 6 and 18 months of age, wherein infants tend to disregard or fail to learn effectively from video-based content. In general, young children learn significantly less from video presentations compared to live, face-to-face interactions. However, emerging evidence suggests that learning from videos can be improved when elements of live social interaction, such as eye contact, joint attention, or responsive communication are incorporated [42].

Research has established that even though 15-month-olds are able to acquire simple skills through an interactive app, they do not succeed in transferring that to real-life situations [43]. As well as this, 2.5–3-year-olds have the problem of transferring app-based learning to the real world. But a study by Tarasuik et al. [44] found that 4–6-year-olds, in contrast to the younger ones, succeed in moving skills learned through the app to the real-life setting and learn as much through an app as through physical demonstrations. Surprisingly, there is a counterexample to this pattern; in a study by Archer et al. [45], younger children might be better able to maximize the carryover of learning through an app when an adult user goes through the experience together with the child and offers personalized support and assistance. Subtle involvement of the adults also seems important for 2.5–3-year-olds, as observed by Eisen et al. [46], which demonstrate improved learning transfer when adults are covertly engaged. These results also carry across to 5–6-year-olds in their evidence that an adult’s presence still leads to more effective app-based learning [47,48].

Some studies have highlighted the multifaceted impact of electronic device use across different developmental stages, emphasizing the need for age-specific considerations between children and adolescents [9,10]. Indeed, Arnett J.J. [10] underscored how adolescents actively use media for self-socialization, shaping their identity and social behaviors, which makes understanding media exposure during adolescence crucial. More recently, Arnett C.P. [9] raised concerns about the digital confinement of children. Specifically the authors criticize excessive digital exposure as a form of “digital confinement” that restricts freedom and natural development.

## 6. Language Ability Development

The foundational work of Stephen Krashen [13] and Eric Lenneberg [12] offers valuable theoretical lenses through which we can interpret language development in the digital era, particularly in children. Krashen’s Monitor Model, especially his “Input Hypothesis and Affective Filter Hypothesis” [13] remains highly relevant: (i) digital media and comprehensible input: Krashen emphasized that language is best acquired through meaningful, understandable input. In the digital age, children are exposed to unprecedented volumes of multimedia language input (e.g., videos, apps, games). If this input is age-appropriate and engaging, it can provide rich linguistic exposure and support second-language acquisition; (ii) affective filter in digital settings: Krashen noted that low stress and high motivation enhance language acquisition. Interactive, gamified digital tools can lower anxiety and increase engagement, potentially improving outcomes if well-designed. However, overstimulation or passive screen use may have the opposite effect; (iii) limits of conscious learning: digital tools focused heavily on grammar drills or formal instruction may align less with Krashen’s model, which favors naturalistic acquisition over rote learning.

Lenneberg’s concept [12] of a critical period for language development (typically considered to end around puberty) has significant implications: (iv) early digital exposure: the critical period theory suggests that the brain is biologically primed to acquire language early in life. In the digital context, excessive screen time, especially passive viewing, could displace rich social interaction and live language exposure, potentially interfering with optimal development during this sensitive window; (v) importance of social interaction: Lenneberg’s view supports the idea that biologically driven mechanisms require interaction and stimulation. Language acquisition depends not just on input, but on human interaction, which some forms of digital media fail to replicate. For instance, passive watching of videos lacks the reciprocal exchange needed for robust language development.

Given these premises, it is not unexpected that a growing number of studies have established a significant correlation between excessive use of devices, such as smartphones, tablets, and televisions, and delays in both expressive and receptive language skills, especially in early childhood.

A large-scale Danish study [49] involving over 31,000 toddlers aged 2 to 3 years found that children who used mobile devices for more than one hour daily had significantly lower expressive and receptive language scores [49]. Similarly, a Canadian study by Madigan et al. [50] correlated mobile media use at 18 months with expressive language delay. Research from Thailand confirmed that more than two hours of daily screen time increases the risk of language delays in toddlers [51]. In particular, 40.9% of a Thai population sample presented suspected language delay and the best predictor was in excess of two hours of screen use per day. Such evidence comes in the form of a systematic review, which pooled results of more than a decade’s worth of studies to conclude that high levels of screen exposure are unfavorably linked to children’s expressive vocabulary and general language skill. Studies conducted in Saudi Arabia and Egypt reported that children who spent many hours per day on smart devices not only showed delayed language skills but also increased scores on social communication screening tools, including attention deficits and hyperactivity [52,53]. Overall, screen exposure in early childhood appears to hinder language development, particularly when it replaces real-world verbal and social engagement [53]. Concrete evidence of the neurostructural alterations related with the language development were observed in a cross-sectional study of children aged 3 to 5 years (*n*  =  47), recruited at a US children’s hospital and community primary care clinics from August 2017 to November 2018 [54]. Children completed cognitive testing followed by diffusion tensor imaging (DTI), and their parent completed a survey on the digital use duration. This study found an association between increased screen-based media use, compared with the AAP guidelines, and lower microstructural integrity of brain white matter tracts supporting language and emergent literacy skills in prekindergarten children [54].

Since reading significantly contributes to language development by expanding vocabulary [13] a study compared in 19 children aged 8–12, the time spent using screen-based media or reading on the functional connectivity of the reading-related brain regions, which strongly influence the language skills [55]. The methodological approach involved parent-reported surveys on the number of hours their children spent on independent reading and screen-based media use, combined with MRI analyses to assess resting-state functional connectivity. Specifically, the analyses focused on the left visual word form area (VWFA) as the seed region and examined its connectivity with other brain regions, using screen time and reading time as predictive variables. The results indicated that greater time spent reading was associated with enhanced brain connectivity, whereas increased exposure to screen-based media was linked to reduced connectivity. Notably, reading and screen time exerted distinct effects on functional connectivity between the VWFA and brain areas involved in language processing, visual perception, and cognitive control [55]. No studies were reported for adolescents.

## 7. Visual Ability

There is still a limited number of studies examining the long-term effects of screen exposure on visual impairments, despite this being one of the most apparent and potentially well-documented areas of impact on visual health. In a review by Wang et al. [56] analyzing 27,110 subjects with a mean age ranging from 9.5 to 26.0 years, researchers found that visual function scores were worse in groups abusing smartphones than in groups with low usage. Suggesting that prolonged smartphone use could increase the likelihood of ocular symptoms, including myopia, asthenopia and ocular surface diseases, especially in children. There is a need to determine the duration of use and restrict prolonged smartphone usage among children and youth. A study released recently by Harrington et al. [57] demonstrated that the prolonged use of electronic screens in children aged 5–6 increases the risk of developing visual disorders such as myopia, hyperopia and higher levels of astigmatism. The prolonged exposure to the artificial light emitted by screens seems to cause early eye strain and a higher rate of visual impairment as previously described [37,38].

## 8. Sleep Hygiene

Systematic review identified, in 90% of the studies, screen use, involving televisions, computers, games consoles, and mobile devices, as being adversely related to preschool, school-aged child and adolescent sleep outcomes, specifically in the form of lower night-time duration and delayed bedtime [4,6,9,10,58,59].

Sleep patterns, a critical factor for cognitive development, have also been linked to media use. Figueiredo et al. [6] reported increased sleep disturbances and psychological issues during the COVID-19 pandemic, partly attributed to prolonged device use. In another study, Figueiredo et al. [6] emphasized the negative impact of poor sleep and co-sleeping on fluid intelligence and academic performance, linking sleep disruptions, often tied to screen overuse, to cognitive vulnerability on school-aged children (aged 7 to 11) as described in Section 5. Verma et al. [4] provided a biological perspective by connecting excessive screen time with neuroinflammation, which may underlie cognitive and behavioral impairments across different age-groups, preschool, school-aged and adolescence. Additional findings were sustained using an epidemiologically-informed school-based recruitment strategy, which recruited over 11,000 U.S. children age 9–10 across 21 study sites [60]. Greater use of screen media was not just associated with longer sleep onset latency and shorter sleep duration, but also increased severity of multiple types of sleep-wake disturbances [60].

These outcomes are primarily induced by the emission of the blue light of the screen, which inhibits melatonin secretion and affects the circadian rhythm to cause sleep disorganization [61]. Research also shows that disrupted sleep patterns can be linked to mental health issues. One study involving adolescents aged 14–18 found associations between poor sleep and increased symptoms of depression, pre-existing psychological disorders, and suicidal ideation [62]. Adolescents who had later bed times (after midnight) had greater likelihood of depressive symptoms than adolescents who had earlier bed times (earlier than 10 p.m.), which is probably because of less overall amount of sleep and less physical activity that is crucial to regulating sleep. Further, screen use heightens sympathetic arousal and delays the onset of melatonin secretion, disturbing the timing and quality of sleep.

Main findings supporting the association between the screen use (television, computers, computer games, and mobile devices) and some health outcomes related with the altered sleep-wake cycle among preschool, school-aged children and adolescents are summarized below (Table 1).

## 9. Mental and Behavioral Well-Being

The increasing use of smartphones and digital devices among children and adolescents has raised growing concerns about their potential impact on mental health and behavior. For instance, during the COVID-19 pandemic, the screen time among youth aged 0 to 21 rose dramatically from 2.67 to 4.38 h per day, with the most pronounced increases observed in older children and adolescents, underlining the widespread and age-transcending nature of this trend [6,48]. In the following subparagraphs, we will focus specifically on two key areas of psychological impact caused by digital device use: depression and emotional dysregulation. These domains have shown consistent associations with excessive screen time and will be examined in detail across different developmental stages.

### 9.1. Depression

Electronic devices are now virtually ubiquitous in the daily lives of many families. Current research supports the hypothesis that there are connections between the excessive use of electronic devices and the psychological development of pre-school children. Firstly, with regard to emotional development, pre-school children who make excessive use of electronic devices may impair the quantity and quality of sleep (as mentioned previously, Section 8), leading to varying levels of anxiety and depression.

The first study to examine depressive symptoms in early childhood within the context of screen exposure was the National Children’s Study, a U.S. multicenter epidemiological investigation of environmental influences on child health and development [63]. Between 2010 and 2012, the study followed 2152 children, evaluating perinatal and demographic variables. Findings revealed that increased exposure to television and/or video content, along with reduced caregiver–child interactive play at 12 months of age, were each significantly associated with greater Autism Spectrum Disorders (ASD)-like symptoms—measured by total scores on the Modified Checklist for Autism in Toddlers revised (M-CHAT-R)—though not with a formal ASD diagnosis. Conversely, lower levels of screen exposure and higher levels of parent–child play at 12 months were associated with fewer ASD-like symptoms at age 2. The study emphasizes the importance of early experiential factors and calls for further research into how these variables influence socio-emotional development [63].

A study by Ranum et al. [64] suggested a correlation between sleep deprivation in 6- and 8-year-old children and the onset of disorders such as anxiety and depression in the following two years, thus pointing to a bidirectionality between sleep quality and the presence of depression and anxiety. Additionally, inadequate sleep has been shown to mediate aggressive behavior and increase vulnerability and depression in adolescents, especially in school contexts where long school hours and poor rest were linked to higher levels of bullying and disruptive behavior [6]. Secondly, the various contents of electronic media, including acts of violence, rape, and murder, may be a risk factor affecting children’s peer relationships. There is concern that exposure to violent digital content may be potentially associated with the risk for poor interpersonal relationships and antisocial behavior [65]. Such content can also trigger psychoneurological effects, as it may contribute to excessive and potentially addictive screen use, leading to reduced social coping abilities and the development of craving-like behaviors that resemble those seen in substance dependence [65]. This could also be a source of family tension due to negative emotional control and conflict between parents and children caused by electronic devices, contributing to increased vulnerability on a psychological level [66,67].

Data from the Adolescent Brain Cognitive Development (ABCD) study identified specific patterns of brain development associated with internalizing problems linked to frequent screen time in a cohort of 5166 participants aged 9–10 years [68]. In a two-year follow-up using structural imaging data, the co-development analysis revealed that the rates of change in gray matter volume within the brainstem, as well as in gray matter volume and cortical thickness of bilateral superior frontal, rostral middle frontal, inferior parietal, and inferior temporal regions, were more closely aligned with one another than with other brain areas. Notably, this association appeared to be mediated by cortical–brainstem circuitry, although the observed effect sizes were relatively small [68].

Furthermore, a large-scale representative sample of UK adolescents (13–15 years) investigated associations between time spent on social media and depressive symptoms adopting the recording in time use diaries. These findings provided that girls tend to be more vulnerable to manifest depression and lower self-esteem due to the greater use of social media [69]. Another evidence further highlighted that adolescents (10–16 years) may daily spend excessive screen-time and are more likely to experience a range of school, behavioral, and mental health difficulties (SBMDs) [70]. These include poor academic performance, obesity, use of substances such as alcohol, tobacco, cannabis, or other illicit drugs, experiences of violence or sexual abuse, perpetration of violence, low social support, depressive symptoms, and suicide attempts. While socioeconomic adversity contributes to these outcomes, its role appears to be modest in the association between daily screen time and SBMDs [70]. Interestingly, as demonstrated in a population-level study [71], mental health outcomes, specifically symptoms of depression and anxiety, improved among adolescents who engaged in extracurricular activities and had lower screen time. The study followed a cohort of 28,712 students who entered seventh grade, tracking their screen use and mental health over a four-year period (from September 2012 to September 2018) through annual surveys. The findings underscore the protective role of structured, real-world engagement in mitigating the negative psychological effects associated with prolonged screen exposure [71].

### 9.2. Social-Emotional Dysregulation

Absorption in games and digital content could replace face-to-face interactions, negatively affecting the development of social skills [72]. Alongside reduced peer-to-peer interaction time there also exists reduced parent–child interaction in the form of reading together and interactive play using toys, which in turn also reduces verbal interaction opportunities with the parent. Reduced parent–child verbal interaction has been linked to negative developmental outcomes as a function of language attainment, self-regulation and academic achievement in the future [67].

A more recent study by Gath et al. [73] also showed a possible correlation between the excessive use of electronic devices and a decrease in time spent in direct social contact in 84 children aged 3–5 years. A study by Hosokawa et al. [72] analyzing 1642 children aged 6, found a risk associated with the routine and frequent use of mobile devices, including smartphones and tablets, by children, which is linked to emotional/behavioral problems. Specifically, it suggests how the use of devices (smartphones, computers) drives children to social isolation, leading to depression and loneliness.

Another study also suggested these trends [74]. In fact, out of a total of 399 participants aged between 2 and 12 years, 53.2% of children who used mobile phones were associated with behavioral problems such as isolation, thus avoiding social encounters, and 39.9% demonstrated rude behavior towards their parents in aspects concerning social interaction and behavior [74]. Similarly, institutional and educational environments lacking adequate emotional support have been associated with difficulties in attention, emotional regulation, and sleep in children and adolescents, highlighting the role of contextual and systemic factors in shaping digital vulnerability as demonstrated by [6].

Here, the focus is on the mood and behavior alteration caused by the sleep deprivation beyond the well-known effects it can have on the physical health, as previously discussed in Section 3. As previously evaluated by these authors, the emotional instability and intellectual performance of children is much more likely correlated with the sleep hygiene within the household [75]. This investigation involved 46 Portuguese children aged 7 to 11, with varying chronotypes and categorized as either ‘only children’ or having siblings. Their results suggest that the co-sleeping—more prominent among children with siblings—are associated with enhanced intellectual development [75]. Building on this work, the authors now turn to the school environment as a critical setting for fostering socio-emotional skills [76]. Here, children often spend more time than at home, participating in extracurricular activities. Therefore, they explore in 117 students in the second and third grades of elementary school, an unusual but meaningful link that may help develop new predictive indicators: the correlation between sleep problems and the tendency to be bullied [77]. Their analysis confirmed that children who reported more episodes of victimization also experienced greater sleep difficulties.

Although these studies are valuable, it is important to highlight that the analyses carried were not specifically designed to collect, track, and monitor mobile device usage and sleep duration in relation to their more detrimental impact on emotional regulation. Aside from the thorough review of Choi et al. [78], which examined sleep disturbances among the adverse effects of screen time, most studies lack rigorous methodological procedures. In particular, the absence of targeted investigation, such as specific assessment of bedtime media use, often leads to contradictory or inconsistent findings. That is what Choi et al. [79] claim sustaining that digital tools can also have therapeutic potential for managing mood disorders. Game-based digital interventions such as *EndeavorRx* or *SPARX* have shown sustained improvements in attention, emotion regulation, and depressive symptoms in children and adolescents, especially in those with neurodevelopmental conditions [79].

## 10. Lifestyle and Physical Activity

Several observational studies suggested correlations between exposure to electronic media and an increase in the risk of obesity. Randomised controlled trials that have attempted to restrict viewing in public have been found to restrict weight gain in children, confirming a cause-and-effect relationship. Current evidence suggests that exposure to screened media leads to obesity in children and adolescents (10- to 15-year-olds) through increased food intake during viewing, exposure to advertisements for high-calorie, low nutritional-value foods and beverages that influence children’s preferences, purchase requests and eating habits [80]. Furthermore, sedentary lifestyles exacerbated by excessive screen use, adversely affect cognitive function, brain structures and abilities [81]. According to Mongkonkansai et al. [82], smartphone ownership, duration of smartphone use, parental rules on smartphone use and posture are associated with musculoskeletal symptoms in 233 children attending primary school. Using smartphones in a prone position presents a higher risk of developing musculoskeletal disorders related to ergonomia. Those who use smartphones for more than 60 min a day are 10 times more likely to develop musculoskeletal disorders than students who use them for less than 60 min. According to rules on smartphone use set by parents, students without rules on smartphone use have six times more musculoskeletal pain than those who follow a rule on smartphone use [82].

## 11. Discussion

The age of digital natives represents a critical factor for investigating the biological effects occurring during development (i.e., early and middle childhood, adolescence), allowing for a more accurate and stratified assessment of the health consequences associated with prolonged digital screen use [83]. Our findings revealed that the effects of device use on neuroplasticity and neurodevelopment are not yet sufficiently explored in middle childhood and adolescence compared to early and preschool years. Epigenetic correlation with techno-stress was investigated in children by Burgio [11]. Other contributions are not developed to better study age-differences and the number of potential molecular markers influenced by the screen use during the developmental stage. The exception is a recent work on university students but this is outside our field of interest as it targets the young adults [84].

In particular, the digital anticipation and digital addiction seem to be strongly correlated with socio-demographic condition such as the working parents [5]. Parents feel ill equipped to help their children navigate such a complex environment. For some children, smartphone ownership starts even sooner as young as 7 years, analyzed the evidence on media use and its long-term consequences in adolescence [85]. Obesity, distraction, addiction, cyberbullying, and the Hikikomori phenomena are described in adolescents who use media device too frequently [85]. It is worth mentioning a study that examined prolonged device use in 400 Canadian adolescents, both with and without autism, in order to distinguish its impact on behavior and performance [86]. Although significant, this study focuses on youth with psychocognitive vulnerabilities—a group that, while not outside the scope of our initial focus, represents a distinct and important subgroup. There remains a lack of studies highlighting the risks faced by individuals with preexisting vulnerabilities, who may be more susceptible to online dangers [34] and the development of internalizing and externalizing problems [86].

The findings may help delineate processes contributing to internalizing behaviors and assist in identifying individuals at greater risk for such problems [68]. Notably, most of the positive findings related to device use come from studies involving children or adolescents with special needs or motor and cognitive impairments, where successful outcomes largely depend on how the technology is used and administered. These studies consistently report high efficacy, likely because device use in these contexts is closely monitored and directed toward specific improvement goals through apps, video games, and virtual reality [87,88].

Therefore, the present review highlights the necessity for further research to elucidate the causal relationships and underlying mechanisms linking screen time and neurological correlates encompassing physical, cognitive, emotional, and behavioral functions, thereby informing guidelines for healthy media consumption [4]. It is also needed to determine the optimal amount and type of screen time that is beneficial for child development. Healthcare providers and parents should be aware of the potential risks of excessive screen time and implement strategies to minimize this exposure [89]. The Italian Pediatric Society provide action-oriented recommendations for families and clinicians to avoid negative outcomes [85]. These advices on screen-based media use are in line with the limits proposed by AAP [8] as follows:

- Infants (under 18–24 months): Avoid all screen time except for video chatting with a caregiver, as it’s not beneficial for development in this age group.
- Toddlers (2–5 years): Limit screen time to 1 h per day of high-quality educational programming, and encourage interactive experiences with caregivers.
- Older Children and Adolescents: The AAP encourages a “Family Media Use Plan” to establish healthy habits, focusing on balance, content, co-viewing, and open communication.

Moreover, AAP advice includes:

- Avoiding screen time as a substitute for interaction, play, or sleep, according to the AAP.
- Creating media-free zones and times (e.g., bedrooms, mealtimes).
- Being mindful of your own media use as a role model, according to the AAP.
- Choosing high-quality content that is age-appropriate, educational, and engaging.
- Co-viewing or co-playing with children to help them understand and learn from media.

In the context of digital age language development, several key implications emerge from established theoretical frameworks. High-quality digital content, such as interactive storybooks and language-rich educational games, may support language acquisition by providing meaningful, comprehensible input, aligning well with Krashen’s model of second language learning. However, excessive passive screen time, especially during early childhood, may interfere with the critical social and neurological foundations required for robust language development, as suggested by Lenneberg’s theory of a sensitive period. Therefore, the most developmentally supportive approach is a balanced one: integrating digital tools with rich, real-world human interaction to optimize both cognitive engagement and linguistic growth.

### 11.1. Early Childhood (0–5 Years)

Evidence suggests that screen exposure in early childhood can affect core competencies necessary for self-regulation and learning. A neuroimaging study has shown that young children aged about 3 who are very exposed to screens are poorer at inhibitory control, an essential function for the regulation of behavior [40]. These impairments are linked to lower activation of the prefrontal cortex, which has led to the suggestion that an overload of screen exposure during the period of increased plasticity is hindering normal neural development. Further investigations involving children aged 3 to 6 [90]. Reestablished that excessive screen use is associated with raised levels of the stress biomarker cortisol as well as with lower academic performance and poorer language skills. These impacts were most evident in less advantaged populations, including refugee groups and those who are developmentally delayed. In these situations, screen use may be replacing critical parent-verbal child interaction on which language and socio-emotional growth are built.

### 11.2. Middle Childhood (6–11 Years)

During middle childhood, these effects are not only on cognition but extend into the emotional and behavioral realms as well. A neuroimaging analysis in Singapore found that excessive early-life screen use is related to accelerated hyper-integration between the cognitive and emotional brain networks at the age of 6. This hyper-integration is hypothesised to be a maladaptive maturity advancement associated with impaired socio-emotional functioning at the age of 7 [91]. However, parental behaviors such as shared reading were found to have a protective role, attenuating the adverse neurodevelopmental effects of screen time. Additional findings from European cohorts [90] indicate that kids who spend more than two hours a day on the screen do worse on tests of learning, but only when the IQ is not affected directly. These impairments are more associated with attention and stress management deficits than with overall cognitive decline [92].

### 11.3. Adolescence (12–18 Years)

In adolescence, screen use is profoundly interconnected with more complicated psychopathological dynamics. Results from the ABCD Study, one of the largest longitudinal brain development samples in the United States documented an interactive relationship between screen use, disrupted sleep, and psychological symptomology including anxiety, depression, and behavioral impulsivity [93]. At early ages 9–10, screen use is predictive of disrupted sleep one year later, which in turn accelerates internalizing (for example, anxiety) and externalizing (for instance, aggression) symptoms [93]. Neurostructural research has found certain brain patterns between the thalamus, prefrontal cortex, and brainstem to be associated with greater susceptibility to behavioral disorders and screen addiction in the youth population. These structural changes can interfere with emotion regulation pathways and impulse management [4]. More recently, excessive screen time has furthermore also been associated with persistent neuroinflammatory conditions during adolescence, primarily because of circadian rhythm disorder and prolonged activation of the stress system. These inflammatory responses constitute a biological substratum for anxiety, mood disorders, and impaired cognition.

To address some of the potential negative clinical outcomes, Table 2 also summarizes practical recommendations aimed at mitigating these effects.

## 12. Limits of the Study and Research Gaps

This review presents some limitations that should be acknowledged. The literature concerning the neurobiological effects of the digital device exposure in adolescents is relatively limited compared to that on younger children, particularly those in the preschool age range. This disparity is partly attributable to a range of research gaps. One of the most significant factors is that the use of mobile devices and internet access is less restricted for teenagers due to increased autonomy and the normalization of certain safety-related practices, such as family tracking and GPS activation, that have become routine. Additionally, smartphones are now essential tools for transportation, mobile payments, and educational purposes, beyond their role in maintaining social connections and communication with family and friends. Another main gap is that the focus of many studies on early developmental stages where motor and cognitive skills are rapidly evolving and more vulnerable to environmental influences.

Consequently, our review not only placed greater emphasis on the younger age groups —where evidence is undoubtedly more abundant—while also striving to capture and encompass all critical periods of neurodevelopment up to adolescence. The lack of uniformity and clarity across the available studies, particularly regarding sample characteristics, types of intervention, study design, follow-up, and inclusion criteria precludes the use of standardized risk of bias (RoB) assessment tools, such as those developed by the Joanna Briggs Institute (JBI) and the Cochrane Collaboration.

Future research should also examine the direction of the associations with self-harm and other mental health outcomes and explore gender differences in how adolescents engage with social media as well as how much time they spend online as remarkably suggested by [69].

## 13. Conclusions

Screen exposure in children and adolescents has age-specific effects, yet a unifying concern emerges: the substitution of relational, physical, and exploratory activities with digital content appears to disrupt physiological rhythms, brain connectivity, and emotional development. In early childhood, this manifests as delayed inhibitory control and impaired language acquisition; in school-aged children, it undermines emotional regulation and attention; and in adolescents, it is associated with neurostructural changes and heightened vulnerability to psychiatric symptoms. Despite these risks, the family environment—particularly the quality of parent–child interactions and practices such as shared reading—can serve as a powerful protective factor. These findings underscore the urgent need for context-sensitive, developmentally tailored strategies to support healthy digital engagement. Ultimately, as we navigate the growing presence of technology in children’s lives, a balanced and vigilant approach is essential. Without thoughtful management, digital media has the potential to compromise key aspects of child and adolescent well-being, demanding serious attention in both research and practice.

According to the International Agency for Research on Cancer (IARC) evaluation of carcinogenic risks to humans and the WHO, the potential health effects of RF-EMR remains undetermined in cancer [94] while confirm the possibly increased vulnerability to neurodegenerative conditions [30].

Crucially, parent–child reading together in the 3rd year showed a protective role: children who partook in more reading activities manifested decreased screen-related changes in brain network integration. This implies that interactive and relational activities, such as parent–child reading together, may protect against the effect of passive digital exposure on the developing brain [41]. Therefore, it’s essential to strike a healthy balance, ensuring children engage in a variety of activities including outdoor play and hands-on experiences that promote both cognitive and physical development [26].

## Figures and Tables

**Table 1:** Childhood health implications associated with the nighttime use of digital screens.

Health Problem	Description	Age Group	Reference
Reduced sleep duration	Screen use is associated with shorter night-time sleep.	Preschool age, Middle childhood, Adolescence	[6]
Delayed bedtime	Exposure to screens leads to a shift in sleep onset time.	Preschool age, Middle childhood, Adolescence	[6]
Neuroinflammation	Excessive screen time trigger neuroinflammation, impacting behavior and cognition.	Preschool age, Middle childhood, Adolescence	[4]
Circadian rhythm disruption	Blue light from screens suppresses melatonin secretion, interfering with circadian timing.	Preschool age	[61]
Pandemic-related psychological stress	Excessive screen use during lockdown contributed to sleep issues and psychological distress.	Middle childhood, Adolescence	[6]
Social disparities in screen exposure	Highlights systemic inequalities and critical perspectives on compulsory digital use in education.	Middle childhood, Adolescence	[9]
Sleep disturbances	Greater screen media use linked to more severe sleep-wake disturbances.	Middle childhood	[60]
Reduced fluid intelligence	Sleep disturbances and co-sleeping are associated with lower cognitive performance and academic vulnerability.	Middle childhood	[6]
Depression and suicidal ideation	Poor sleep patterns linked to depressive symptoms, psychological distress, and suicidal ideation.	Adolescence	[62]
Reduced physical activity	Evening screen use reduces sleep duration and limit necessary physical activity for proper sleep regulation.	Adolescence	[62]
Increased sympathetic arousal	Screen exposure increases sympathetic arousal and delays melatonin onset, impairing sleep quality.	Adolescence	[62]
Media self-socialization	Media used for self-socialization affect sleep routines.	Adolescence	[10]

**Table 2:** Impact of screen time on neurodevelopment domains across age groups.

Section	Key Findings	Age Group			Recommendation	Ref.
		Early Childhood (0–5 Years)	Middle Childhood (6–11 Years)	Adolescence (12–18 Years)		
**3. Motor Ability**	Multiple studies reveal both benefits and risks of digital device use on motor development across age groups. In early childhood, supervised use of educational apps can enhance fine motor control, writing skills, and visuospatial coordination. However, excessive or unsupervised screen time is linked to delays in motor development, poor posture, and reduced physical activity. In middle childhood, findings are mixed—while gamified digital tasks may support fine motor skills, sedentary behavior remains a concern. Among adolescents, evidence is limited and mostly indirect, but high screen use is consistently associated with decreased physical activity and potential negative health effects.	**✓**	**✓**	**✓**	Encourage supervised interactive screen use; limit passive or excessive screen time especially in preschoolers; promote outdoor physical play.	[2,3,5,19,20,20,21,22,23,24,25,26,27,28,29]
**4. Cognitive and Behavioral Ability**	Excessive screen use in early and middle childhood is linked to altered stress responses, reduced inhibitory control, and emotional dysregulation. Neuroinflammation and oxidative stress from RF-EMR may disrupt brain development, leading to anxiety, behavioral issues, and attention problems. Adolescents face similar risks, including social-emotional maladjustment.	**✓**	**✓**	**✓**	Educate parents on exposure; limit unsupervised screen time; implement digital detox routines during key developmental windows.	[11,27,30,31,32,33,34,35,36,37,38,39,40,41]
**5. Learning and Memory Ability**	Younger children, particularly under 3, exhibit a ‘video deficit’, learning less from screen-based content compared to face-to-face interaction. Older preschoolers (4–6 years) may successfully transfer knowledge from digital tools when supported by adult engagement. Middle childhood benefits from structured learning apps, while adolescence sees limited evidence of cognitive benefits without active guidance.	**✓**	**✓**	**✓**	Foster active co-viewing; select educational apps that encourage real-world problem-solving; avoid replacing direct teaching with digital media in early years.	[42,43,44,45,46,47,48]
**6. Language Ability Development**	Heavy screen use in toddlers is strongly associated with expressive and receptive language delays. Optimal development depends on live verbal interaction, which is compromised by passive viewing. Neuroimaging studies show reduced white matter integrity in children with high screen exposure. Middle childhood shows altered reading-brain connectivity, while adolescent data are lacking.	**✓**	**✓**	**X**	Limit screen time to <1 h/day in toddlers; prioritize interactive language play and shared book reading; avoid using devices as substitutes for conversation.	[49,50,51,52,53,54,55]
**7. Visual Ability**	Prolonged digital screen use, particularly on smartphones and tablets, increases the risk of visual problems such as myopia, astigmatism, and eye strain. These effects are more pronounced in younger children but extend into school-aged populations. Studies warn against excessive screen time without breaks.	**✓**	**✓**	**✓**	Introduce screen breaks every 20–30 min; use natural lighting and ergonomic posture; discourage prolonged exposure in low-light settings.	[37,38,56,57]
**8. Sleep Hygiene**	Screen use, particularly before bed time, disrupts circadian rhythms via melatonin suppression from blue light. Consequences include sleep onset delay, reduced duration, poor sleep quality, and downstream cognitive-emotional problems. These effects are well-documented across all age groups.	**✓**	**✓**	**✓**	Avoid screens 1–2 h before bed; use blue-light filters; encourage physical activity during the day; implement calming bedtime routines.	[6,10,60,61,62]
**9. Mental and Behavioral Well-Being**	Increased screen time is associated with higher risks of depression, anxiety, and behavioral issues in all age groups. Children exposed to violent or overstimulating content may show social withdrawal and impaired emotion regulation. Adolescents are particularly vulnerable to screen addiction and internalizing symptoms.	**✓**	**✓**	**✓**	Monitor content exposure; encourage offline peer interaction; use digital tools therapeutically under professional guidance.	[48,63,64,65,66,67,68,69,70,71,72,73,74,75,76,77,78,79]
**10. Lifestyle and Physical Activity**	Higher screen use correlates with lower physical activity, increased food intake, and risk of obesity, especially when viewing is combined with snacking and ad exposure. Prolonged smartphone use is also linked to musculoskeletal disorders due to poor posture, particularly in middle childhood and adolescence.	**✓**	**✓**	**✓**	Set screen time limits; promote daily physical activity; educate children on proper device posture; restrict media consumption during meals.	[80,81,82]

The symbols **✓** and **X** indicate the presence or absence of evidence, respectively.

## Data Availability

Not applicable.

## References

[1] Auxier B., Anderson M., Perrin A., Turner E. (2020). Parenting Children in the Age of Screens.

[2] Bacil E.D.A., da Silva M.P., Martins R.V., da Costa C.G., de Campos W. (2024). Exposure to Smartphones and Tablets, Physical Activity and Sleep in Children From 5 to 10 Years Old: A Systematic Review and Meta-Analysis. Am. J. Health Promot..

[3] Swider-Cios E., Vermeij A., Sitskoorn M.M. (2023). Young Children and Screen-Based Media: The Impact on Cognitive and Socioemotional Development and the Importance of Parental Mediation. Cogn. Dev..

[4] Verma A., Kumar A., Chauhan S., Sharma N., Kalani A., Gupta P.C. (2025). Interconnections of Screen Time with Neuroinflammation. Mol. Cell. Biochem..

[5] Operto F.F., Viggiano A., Perfetto A., Citro G., Olivieri M., Simone V.d.e., Bonuccelli A., Orsini A., Aiello S., Coppola G. (2023). Digital Devices Use and Fine Motor Skills in Children between 3–6 Years. Children.

[6] Figueiredo S., João R., Alho L., Hipólito J. (2022). Psychological Research on Sleep Problems and Adjustment of Working Hours during Teleworking in the COVID-19 Pandemic: An Exploratory Study. Int. J. Environ. Res. Public Health.

[7] Guidelines on Physical Activity, Sedentary Behaviour and Sleep for Children Under 5 Years of Age. https://www.who.int/publications/i/item/9789241550536.

[8] Council on Communications and Media (2016). Media and Young Minds. Pediatrics.

[9] Arnett C. (2024). No Child Left Confined: Challenging the Digital Convict Lease. J. Health Care Law Policy.

[10] Arnett J.J. (1995). Adolescents’ Uses of Media for Self-Socialization. J. Youth Adolesc..

[11] Sage C., Burgio E. (2018). Electromagnetic Fields, Pulsed Radiofrequency Radiation, and Epigenetics: How Wireless Technologies May Affect Childhood Development. Child Dev..

[12] Lenneberg E.H. (1967). The Biological Foundations of Language. Hosp. Pract..

[13] Krashen S.D. (1985). Second Language Acquisition and Second Language Learning.

[14] Szyf M., Weaver I.C.G., Champagne F.A., Diorio J., Meaney M.J. (2005). Maternal Programming of Steroid Receptor Expression and Phenotype through DNA Methylation in the Rat. Front. Neuroendocrinol..

[15] Diamond A. (2000). Close Interrelation of Motor Development and Cognitive Development and of the Cerebellum and Prefrontal Cortex. Child Dev..

[16] Lupien S.J., McEwen B.S., Gunnar M.R., Heim C. (2009). Effects of Stress throughout the Lifespan on the Brain, Behaviour and Cognition. Nat. Rev. Neurosci..

[17] Binder D.K., Scharfman H.E. (2004). Brain-Derived Neurotrophic Factor. Growth Factors.

[18] Shonkoff J.P., Phillips D.A., National Research Council (US), Institute of Medicine (US) Committee on Integrating the Science of Early Childhood Development (2000). From Neurons to Neighborhoods: The Science of Early Childhood Development.

[19] Courage M.L., Frizzell L.M., Walsh C.S., Smith M. (2021). Toddlers Using Tablets: They Engage, Play, and Learn. Front. Psychol..

[20] Bonneton-Botté N., Fleury S., Girard N., Le Magadou M., Cherbonnier A., Renault M., Anquetil E., Jamet E. (2020). Can Tablet Apps Support the Learning of Handwriting? An Investigation of Learning Outcomes in Kindergarten Classroom. Comput. Educ..

[21] Kostyrka-Allchorne K., Cooper N.R., Simpson A. (2017). The Relationship between Television Exposure and Children’s Cognition and Behaviour: A Systematic Review. Dev. Rev..

[22] Webb S.J., Howard W., Garrison M., Corrigan S., Quinata S., Taylor L., Christakis D.A. (2024). Mobile Media Content Exposure and Toddlers’ Responses to Attention Prompts and Behavioral Requests. JAMA Netw. Open.

[23] Levine L.E., Waite B.M., Bowman L.L., Kachinsky K. (2019). Mobile Media Use by Infants and Toddlers. Comput. Hum. Behav..

[24] Lin L.-Y., Cherng R.-J., Chen Y.-J. (2017). Effect of Touch Screen Tablet Use on Fine Motor Development of Young Children. Phys. Occup. Ther. Pediatr..

[25] Mohamed N.M., Kamal H.M., Gharib R.M. (2023). Effect of Touch Screen Devices Use on Fine Motor Skills of Preschool Children. Egypt. J. Hosp. Med..

[26] Arabiat D., Al Jabery M., Robinson S., Whitehead L., Mörelius E. (2023). Interactive Technology Use and Child Development: A Systematic Review. Child Care Health Dev..

[27] Geng S., Wang W., Huang L., Xie J., Williams G.J., Baker C., Du W., Hua J. (2023). Association between Screen Time and Suspected Developmental Coordination Disorder in Preschoolers: A National Population-Based Study in China. Front. Public Health.

[28] Hardi F.A., Goetschius L.G., Tillem S., McLoyd V., Brooks-Gunn J., Boone M., Lopez-Duran N., Mitchell C., Hyde L.W., Monk C.S. (2023). Early Childhood Household Instability, Adolescent Structural Neural Network Architecture, and Young Adulthood Depression: A 21-Year Longitudinal Study. Dev. Cogn. Neurosci..

[29] Haddock A., Ward N., Yu R., O’Dea N. (2022). Positive Effects of Digital Technology Use by Adolescents: A Scoping Review of the Literature. Int. J. Environ. Res. Public Health.

[30] Manwell L.A., Tadros M., Ciccarelli T.M., Eikelboom R. (2022). Digital Dementia in the Internet Generation: Excessive Screen Time during Brain Development Will Increase the Risk of Alzheimer’s Disease and Related Dementias in Adulthood. J. Integr. Neurosci..

[31] Kıvrak E.G., Yurt K.K., Kaplan A.A., Alkan I., Altun G. (2017). Effects of Electromagnetic Fields Exposure on the Antioxidant Defense System. J. Microsc. Ultrastruct..

[32] Narayanan S.N., Jetti R., Kesari K.K., Kumar R.S., Nayak S.B., Bhat P.G. (2019). Radiofrequency Electromagnetic Radiation-Induced Behavioral Changes and Their Possible Basis. Environ. Sci. Pollut. Res. Int..

[33] Zheng R., Zhang X., Gao Y., Gao D., Gong W., Zhang C., Dong G., Li Z. (2023). Biological Effects of Exposure to 2650 MHz Electromagnetic Radiation on the Behavior, Learning, and Memory of Mice. Brain Behav..

[34] Parano E., Pavone V., Ruggieri M., Castagnola I., Ettore G., Fusto G., Rizzo R., Pavone P. (2025). The Many Faces of Child Abuse: How Clinical, Genetic and Epigenetic Correlates Help Us See the Full Picture. Children.

[35] Pappalardo X.G., Testa G., Pellitteri R., Dell’Albani P., Rodolico M., Pavone V., Parano E. (2023). Early Life Stress (ELS) Effects on Fetal and Adult Bone Development. Children.

[36] Sudan M., Olsen J., Arah O.A., Obel C., Kheifets L. (2016). Prospective Cohort Analysis of Cellphone Use and Emotional and Behavioural Difficulties in Children. J. Epidemiol. Community Health.

[37] Haghani M., Abbasi S., Abdoli L., Shams S.F., Baha’addini Baigy Zarandi B.F., Shokrpour N., Jahromizadeh A., Mortazavi S.A., Mortazavi S.M.J. (2024). Blue Light and Digital Screens Revisited: A New Look at Blue Light from the Vision Quality, Circadian Rhythm and Cognitive Functions Perspective. J. Biomed. Phys. Eng..

[38] Gallego-Rentero M., López Sánchez A., Nicolás-Morala J., Alcaraz-Laso P., Zhang N., Juarranz Á., González S., Carrasco E. (2024). The Effect of Fernblock^®^ in Preventing Blue-Light-Induced Oxidative Stress and Cellular Damage in Retinal Pigment Epithelial Cells Is Associated with NRF2 Induction. Photochem. Photobiol. Sci..

[39] Bonilla P., McBride D., Shanks A., Kartik J., Bhan A., Porcu A. (2025). Evening Blue Light Exposure during Adolescence Induces Avoidance Behaviors and Rewires Medial Amygdala Circuit. bioRxiv.

[40] Meng X., Liang X., Liu C., Cheng N., Lu S., Zhang K., Yin Y., Cheng T., Lu C., Wang Z. (2024). Associations between Screen Media Use and Young Children’s Inhibitory Control: Evidence from Behavioral and fNIRS Study. Comput. Hum. Behav..

[41] Huang P., Chan S.Y., Ngoh Z.M., Ong Z.Y., Low X.Z., Law E.C., Gluckman P.D., Kee M.Z.L., Fortier M.V., Chong Y.S. (2024). Screen Time, Brain Network Development and Socio-Emotional Competence in Childhood: Moderation of Associations by Parent-Child Reading. Psychol. Med..

[42] Capparini C., To M.P.S., Reid V.M. (2023). Should I Follow Your Virtual Gaze? Infants’ Gaze Following over Video Call. J. Exp. Child Psychol..

[43] Zack E., Barr R., Gerhardstein P., Dickerson K., Meltzoff A.N. (2009). Infant Imitation from Television Using Novel Touch Screen Technology. Br. J. Dev. Psychol..

[44] Tarasuik J., Demaria A., Kaufman J. (2017). Transfer of Problem Solving Skills from Touchscreen to 3D Model by 3- to 6-Year-Olds. Front. Psychol..

[45] Archer K., Wood E., De Pasquale D. (2021). Examining Joint Parent-Child Interactions Involving Infants and Toddlers When Introducing Mobile Technology. Infant Behav. Dev..

[46] Eisen S., Lillard A.S. (2020). Learning from Apps and Objects: The Human Touch. Mind Brain Educ..

[47] Xie H., Peng J., Qin M., Huang X., Tian F., Zhou Z. (2018). Can Touchscreen Devices Be Used to Facilitate Young Children’s Learning? A Meta-Analysis of Touchscreen Learning Effect. Front. Psychol..

[48] Choi K., Kirkorian H.L., Pempek T.A. (2021). Touchscreens for Whom? Working Memory and Age Moderate the Impact of Contingency on Toddlers’ Transfer From Video. Front. Psychol..

[49] Rayce S.B., Okholm G.T., Flensborg-Madsen T. (2024). Mobile Device Screen Time Is Associated with Poorer Language Development among Toddlers: Results from a Large-Scale Survey. BMC Public Health.

[50] Madigan S., McArthur B.A., Anhorn C., Eirich R., Christakis D.A. (2020). Associations Between Screen Use and Child Language Skills: A Systematic Review and Meta-Analysis. JAMA Pediatr..

[51] Rithipukdee N., Kusol K. (2022). Factors Associated with the Suspected Delay in the Language Development of Early Childhood in Southern Thailand. Children.

[52] Alrahili N., Almarshad N.A., Alturki R.Y., Alothaim J.S., Altameem R.M., Alghufaili M.A., Alghamdi A.A., Alageel A.A. (2021). The Association Between Screen Time Exposure and Autism Spectrum Disorder-Like Symptoms in Children. Cureus.

[53] Korres G., Kourklidou M., Sideris G., Bastaki D., Demagkou A., Riga M., Gogoulos P., Nikolopoulos T., Delides A. (2024). Unsupervised Screen Exposure and Poor Language Development: A Scoping Review to Assess Current Evidence and Suggest Priorities for Research. Cureus.

[54] Hutton J.S., Dudley J., Horowitz-Kraus T., DeWitt T., Holland S.K. (2020). Associations Between Screen-Based Media Use and Brain White Matter Integrity in Preschool-Aged Children. JAMA Pediatr..

[55] Horowitz-Kraus T., Hutton J.S. (2018). Brain Connectivity in Children Is Increased by the Time They Spend Reading Books and Decreased by the Length of Exposure to Screen-Based Media. Acta Paediatr..

[56] Wang J., Li M., Zhu D., Cao Y. (2020). Smartphone Overuse and Visual Impairment in Children and Young Adults: Systematic Review and Meta-Analysis. J. Med. Internet Res..

[57] Harrington S., O’Dwyer V. (2023). The Association between Time Spent on Screens and Reading with Myopia, Premyopia and Ocular Biometric and Anthropometric Measures in 6- to 7-Year-Old Schoolchildren in Ireland. Ophthalmic Physiol. Opt..

[58] Nakshine V.S., Thute P., Khatib M.N., Sarkar B. (2022). Increased Screen Time as a Cause of Declining Physical, Psychological Health, and Sleep Patterns: A Literary Review. Cureus.

[59] Hale L., Guan S. (2015). Screen Time and Sleep among School-Aged Children and Adolescents: A Systematic Literature Review. Sleep Med. Rev..

[60] Hisler G.C., Hasler B.P., Franzen P.L., Clark D.B., Twenge J.M. (2020). Screen Media Use and Sleep Disturbance Symptom Severity in Children. Sleep Health.

[61] Lee S.-I., Matsumori K., Nishimura K., Nishimura Y., Ikeda Y., Eto T., Higuchi S. (2018). Melatonin Suppression and Sleepiness in Children Exposed to Blue-Enriched White LED Lighting at Night. Physiol. Rep..

[62] Tymofiyeva O., Yuan J.P., Kidambi R., Huang C.-Y., Henje E., Rubinstein M.L., Jariwala N., Max J.E., Yang T.T., Xu D. (2020). Neural Correlates of Smartphone Dependence in Adolescents. Front. Hum. Neurosci..

[63] Heffler K.F., Sienko D.M., Subedi K., McCann K.A., Bennett D.S. (2020). Association of Early-Life Social and Digital Media Experiences With Development of Autism Spectrum Disorder-Like Symptoms. JAMA Pediatr..

[64] Ranum B.M., Wichstrøm L., Pallesen S., Falch-Madsen J., Halse M., Steinsbekk S. (2019). Association Between Objectively Measured Sleep Duration and Symptoms of Psychiatric Disorders in Middle Childhood. JAMA Netw. Open.

[65] Lissak G. (2018). Adverse Physiological and Psychological Effects of Screen Time on Children and Adolescents: Literature Review and Case Study. Environ. Res..

[66] Xiong Y. (2022). Excessive Electronic Media Use: The Effects on Preschoolers’ Development of Emotion and Social Relationships.

[67] Muppalla S.K., Vuppalapati S., Reddy Pulliahgaru A., Sreenivasulu H. (2023). Effects of Excessive Screen Time on Child Development: An Updated Review and Strategies for Management. Cureus.

[68] Zhao Y., Paulus M.P., Potenza M.N. (2023). Brain Structural Co-Development Is Associated with Internalizing Symptoms Two Years Later in the ABCD Cohort. J. Behav. Addict..

[69] Barthorpe A., Winstone L., Mars B., Moran P. (2020). Is Social Media Screen Time Really Associated with Poor Adolescent Mental Health? A Time Use Diary Study. J. Affect. Disord..

[70] Chau K., Bhattacherjee A., Senapati A., Guillemin F., Chau N. (2022). Association between Screen Time and Cumulating School, Behavior, and Mental Health Difficulties in Early Adolescents: A Population-Based Study. Psychiatry Res..

[71] Boers E., Afzali M.H., Newton N., Conrod P. (2019). Association of Screen Time and Depression in Adolescence. JAMA Pediatr..

[72] Hosokawa R., Katsura T. (2018). Association between Mobile Technology Use and Child Adjustment in Early Elementary School Age. PLoS ONE.

[73] Gath M., McNeill B., Gillon G. (2023). Preschoolers’ Screen Time and Reduced Opportunities for Quality Interaction: Associations with Language Development and Parent-Child Closeness. Curr. Res. Behav. Sci..

[74] Iqbal M., Saeed F., Bham S.Q., Khan M.A., Sharif U.H.A. (2022). Impact of Mobile Phone Use on Health, Behavior and Social Interactions among Children Aged 2–12 Years: Impact of Mobile Phone Use on Health, Behavior and Social Interactions. Pak. Biomed. J..

[75] Figueiredo S. (2022). Sleeping Habits Explaining Academic Vulnerability and Household Influence: Co-Sleeping and the Impact on Children’s Fluid Intelligence. Eur. J. Educ. Res..

[76] Figueiredo S., Silvestre P. (2025). Where Do Our Children Go? Understanding the Impact of Institutionalization on Emotion Regulation, Attention, and Sleep. Children.

[77] Figueiredo S. (2024). Bullying and Victimization in Schools: Causal Relationship between Adolescent Disruptive Behavior, Sleep Schedules, and Extended School Hours. Psychol. Sch..

[78] Choi E.J., King G.K.C., Duerden E.G. (2023). Screen Time in Children and Youth during the Pandemic: A Systematic Review and Meta-Analysis. Glob. Pediatr..

[79] Choi E., Yoon E.-H., Park M.-H. (2022). Game-Based Digital Therapeutics for Children and Adolescents: Their Therapeutic Effects on Mental Health Problems, the Sustainability of the Therapeutic Effects and the Transfer of Cognitive Functions. Front. Psychiatry.

[80] Robinson T.N., Banda J.A., Hale L., Lu A.S., Fleming-Milici F., Calvert S.L., Wartella E. (2017). Screen Media Exposure and Obesity in Children and Adolescents. Pediatrics.

[81] Cui J., Li L., Dong C. (2022). The Associations between Specific-Type Sedentary Behaviors and Cognitive Flexibility in Adolescents. Front. Hum. Neurosci..

[82] Mongkonkansai J., Veerasakul S., Tamrin S.B.M., Madardam U. (2022). Predictors of Musculoskeletal Pain among Primary School Students Using Smartphones in Nakhon Si Thammarat, Thailand. Int. J. Environ. Res. Public. Health.

[83] Maeneja R., Rato J., Ferreira I.S. (2025). How Is the Digital Age Shaping Young Minds? A Rapid Systematic Review of Executive Functions in Children and Adolescents with Exposure to ICT. Children.

[84] Annunzi E., Cannito L., Bellia F., Mercante F., Vismara M., Benatti B., Di Domenico A., Palumbo R., Adriani W., Dell’Osso B. (2023). Mild Internet Use Is Associated with Epigenetic Alterations of Key Neurotransmission Genes in Salivary DNA of Young University Students. Sci. Rep..

[85] Bozzola E., Spina G., Ruggiero M., Vecchio D., Caruso C., Bozzola M., Staiano A.M., Agostiniani R., Del Vecchio A., Banderali G. (2019). Media Use during Adolescence: The Recommendations of the Italian Pediatric Society. Ital. J. Pediatr..

[86] Wallace J., Boers E., Ouellet J., Afzali M.H., Conrod P. (2023). Screen Time, Impulsivity, Neuropsychological Functions and Their Relationship to Growth in Adolescent Attention-Deficit/Hyperactivity Disorder Symptoms. Sci. Rep..

[87] Page Z.E., Barrington S., Edwards J., Barnett L.M. (2017). Do Active Video Games Benefit the Motor Skill Development of Non-Typically Developing Children and Adolescents: A Systematic Review. J. Sci. Med. Sport.

[88] Coutinho F., Bosisio M.-E., Brown E., Rishikof S., Skaf E., Zhang X., Perlman C., Kelly S., Freedin E., Dahan-Oliel N. (2017). Effectiveness of iPad Apps on Visual-Motor Skills among Children with Special Needs between 4y0m-7y11m. Disabil. Rehabil. Assist. Technol..

[89] Goswami P., Parekh V. (2023). The Impact of Screen Time on Child and Adolescent Development: A Review. Int. J. Contemp. Pediatr..

[90] Hahnefeld A., Fink M., Le Beherec S., Baur M.A., Bernhardt K., Mall V. (2024). Correlation of Screen Exposure to Stress, Learning, Cognitive and Language Performance in Children. Eur. Child Adolesc. Psychiatry.

[91] Chen Y.-Y., Yim H., Lee T.-H. (2023). Negative Impact of Daily Screen Use on Inhibitory Control Network in Preadolescence: A Two-Year Follow-up Study. Dev. Cogn. Neurosci..

[92] Paulich K.N., Ross J.M., Lessem J.M., Hewitt J.K. (2021). Screen Time and Early Adolescent Mental Health, Academic, and Social Outcomes in 9- and 10- Year Old Children: Utilizing the Adolescent Brain Cognitive Development ^SM^ (ABCD) Study. PLoS ONE.

[93] Zhao Y., Paulus M.P., Tapert S.F., Bagot K.S., Constable R.T., Yaggi H.K., Redeker N.S., Potenza M.N. (2024). Screen Time, Sleep, Brain Structural Neurobiology, and Sequential Associations with Child and Adolescent Psychopathology: Insights from the ABCD Study. J. Behav. Addict..

[94] Moon J.-H. (2020). Health Effects of Electromagnetic Fields on Children. Clin. Exp. Pediatr..

